# 24-Hour Hypoxia and Pulmonary Hypertension in Patients with Idiopathic Pulmonary Fibrosis

**DOI:** 10.2174/1874306401711010010

**Published:** 2017-05-29

**Authors:** Marcelo P. Rodrigues, Carolina M. Vissoci, Samuel P. Rosa, Sandra B.C. Negreiros

**Affiliations:** 1 Department of Pulmonology, School of Medicine, Universidade de Brasília (UnB), Brasília, DF, Brazil; 2 Student of Medicine. School of Medicine, UnB, Brasília, DF, Brazil; 3 Department of Cardiology, Hospital de Base, Brasília, DF, Brazil

**Keywords:** Hypertension, Pulmonary, Hypoxia, Idiopathic pulmonary fibrosis, Blood gas monitoring, Transcutaneous

## Abstract

**Background:**

The quantification of hypoxia based on resting partial pressure of arterial oxygen (PaO_2_) may underestimate hypoxia related to activities of daily living or sleep and thus not accurately reflect pulmonary hypertension (PH). The aim of the present study was to investigate the association of resting PaO_2_ with percent time of SpO_2_ below 90% (T90) and 88% (T88) in 24 hours. We also evaluated the capacity of hypoxia measures to predict PH in patients with idiopathic pulmonary fibrosis (IPF).

**Method:**

This cross-sectional study included 27 patients with IPF presenting PaO_2_ ≥ 55 mmHg and not receiving home oxygen therapy. All were submitted to blood gas measurement, 24-h oximetry, and transthoracic Doppler echocardiography to estimate systolic pulmonary artery pressure (SPAP). Patients were divided into three groups according to resting PaO_2_: 55-55.9 mmHg (A); 60-60.9 mmHg (B); ≥ 70 mmHg (C). PH was defined as “likely” if SPAP > 50 mmHg, and as possible for SPAP between 37 and 50 mmHg.

**Results:**

T90 and T88 in Groups A, B, and C were as follows: 59.9±29% and 44.1±34%; 49.3±34% and 29.9±31%; 17.1±25% and 8.8±18% respectively, with significant differences between the groups for both T90 (*p* ≤ 0.01) and T88 (*p* = 0.02). PaO_2_ was inversely correlated with T90 (r = -0.398; *p* = 0.04) and T88 (r = -0.351; *p* = 0.07). Hypoxia variables did not correlate with SPAP, and were not able to predict PH.

**Conclusion:**

Percent time of SpO_2_ below 90% and 88% in 24 hours revealed periods of severe hypoxia even in patients with borderline-normal resting PaO_2_. However, none of the present hypoxia variables was capable of predicting PH.

## 1 INTRODUCTION

Pulmonary hypertension (PH) is a common finding in patients with idiopathic pulmonary fibrosis (IPF), and increases in frequency and intensity with the progression of IPF [1]. PH is associated with significant reduction in IPF survival, and thus has important prognostic implications for IPF patients [2, 3].

Traditionally, PH associated with chronic lung diseases has been attributed to alveolar hypoxia. Animal models suggest that chronic alveolar hypoxia causes PH through a combination of sustained vasoconstriction and vascular remodeling. It is generally accepted that vasoconstriction plays a major role during initial stages of the disease, whereas structural remodeling of the pulmonary vascular bed becomes more important during later stages of the disease. This hypothesis is supported by the observation that oxygen administration becomes less effective to reduce pulmonary arterial pressure with increased duration of chronic alveolar hypoxia. The lack of response to oxygen therapy, in turn, suggests that PH results, at least in part, from structural remodeling that is irreversible or only minimally reversible [4]. Nevertheless, there is no consensus on this issue, even in animal models; in rats, Hyvelin *et al.* [5] have shown that sustained vasoconstriction, largely dependent on Rho-kinase action, plays a more important role in the development of hypoxia-induced PH than structural vascular changes.

An aspect that has been overlooked regarding the role of hypoxia in the establishment of PH is the method used to measure the degree of hypoxia. Peripheral capillary oxygen saturation (SpO_2_) often decreases during exercise in patients with IPF. In addition, the severity of intermittent sleep oxygen desaturation has been previously associated with shorter survival in patients with IPF [6]. Thus, quantifying hypoxia based strictly on resting partial pressure of arterial oxygen (PaO_2_) may mask the impact of activities of daily life and sleep.

The present study aimed at correlating resting PaO_2_ with duration of SpO_2_ below 90% (T90) and 88% (T88) in 24 hours (to incorporate the effect of activities of daily living and sleep) in a group of patients with IPF. In addition, we evaluated the ability of different hypoxia measures to predict PH in this sample.

## 2 METHOD

A cross-sectional study was carried out with 27 patients selected consecutively from the Interstitial Lung Disease Clinic at a tertiary referral hospital (Hospital Universitário de Brasília). IPF was diagnosed according to American Thoracic Society/European Respiratory Society criteria [7]. In 10 patients (37%), surgical lung biopsy was required to confirm the diagnosis. In the remaining 17 patients, clinical and radiologic criteria were considered to be sufficient for diagnosis. Additional inclusion criteria were stable clinical status, resting PaO_2_ ≥ 55 mmHg, and not receiving home oxygen therapy. Exclusion criteria were chronic pulmonary thromboembolism and clinical or echocardiographic evidence of left ventricular dysfunction.

The study was approved by the Ethics Committee at Universidade de Brasília. All participants signed an informed consent form.

### 2.1 Spirometry and Determination of PaO_2_ and 24-h SpO_2_

Forced vital capacity (FVC) was determined following American Thoracic Society guidelines [8]. For blood gas measurements, blood was drawn from the non-dominant arm radial artery. Blood samples were collected after a 20-min rest in the sitting position and local anesthesia with 2% lidocaine solution. Blood samples were analyzed in a blood gas analyzer for 3 minutes or less (AVL Compact 3, Graz, Austria). The alveolar gas equation was used to calculate D_A-aO2_ considering a local atmospheric pressure of 680 mmHg, with respiratory quotient of 0.8 for all patients.

For the present study, patients were divided into 3 groups, according to resting PaO_2_: borderline for indication of home oxygen therapy (PaO_2_ 55 to 55.9 mmHg) (Group A); intermediate (PaO_2_ between 60 and 69.9 mmHg) (Group B); and mild hypoxia or normal PaO_2_ (PaO_2 =_ 70 mmHg) (Group C).

Oxygen saturation was measured over 24 hours using a wrist-worn pulse oximeter (Nonin 3100, Minneapolis, USA) attached to the index finger in the non-dominant hand. Patients were instructed to keep their habitual activities. 24-h measurements were analyzed using the nVision software v. 5.1e (Nonin, Minneapolis, USA). The percentage of wake and sleep time with SpO_2_ below 90% (T90) and 88% (T88) was measured in relation to the total 24-h period.

### 2.2 Measurement of SPAP and PH

Transthoracic Doppler echocardiography was performed with patients in the lateral recumbent position using an ultrasound device (General Electric, VIVID S5, Milwaukee, USA) with multi-frequency transducer (2.5 to 3.5 MHz). Standard projections were used.

Chamber quantification and ventricular function assessment were performed as recommended by the American Society of Echocardiography [9]. Real-time Doppler color flow mapping was used to more accurately assess tricuspid regurgitation. Continuous wave Doppler with sweep speed of 50-100 mm/s was used. Three to five measurements were performed per parameter.

SPAP was calculated based on tricuspid regurgitation using the modified Bernoulli equation. The pressure gradient between the right ventricle and right atrium was thus obtained. Estimated right atrial pressure was added to the Doppler-determined gradient [10], considering an unobstructed right ventricular outflow tract. Right atrial pressure estimates were based on inferior vena cava percentage collapse during spontaneous breathing. If inspiratory collapse was greater than 50% and initial diameter was smaller than 2.1 cm, an estimated right atrial pressure of 5 mmHg was added to the Doppler-determined gradient; if the collapse was 50% or more and the initial diameter ≥ 2.1 cm, 10 mmHg were added. In patients with inferior vena cava dilatation to a diameter significantly higher than 2.1 cm and collapse significantly less than 50%, 20 mmHg were added [11-13].

PH was defined as “likely” if SPAP > 50 mmHg [13, 14]. PH was defined as possible for SPAP between 37 and 50 mmHg, or for SPAP below this level in the presence of the following echocardiographic findings: right ventricular dilatation and/or hypertrophy, paradoxical septal motion, or right ventricular dysfunction according to American Society of Echocardiography recommendations for assessment of right ventricle [13].

### 2.3 Statistical Analysis

The distribution of continuous variables was determined using the Kolmogorov-Smirnov test as well as a Q-Q plot, a graphical representation in which normal distribution is represented by a straight diagonal line. Since all variables under study (T90, T88, PaO_2_, D_A-aO2_, SPAP) had normal distribution, group means were compared using one-way analysis of variance (ANOVA). Correlations were evaluated using Pearson’s coefficient. For the binary yes/no variables (likely PH and combined analysis of possible + likely PH) logistic models were employed for each indicator of hypoxia (T90, T88, PaO_2_, D_A-aO2_), and odds ratios were calculated for these predictors with 95% confidence intervals. Means are presented with standard deviations. Significance was defined as *p* < 0.05. SPSS v. 20 for Mac was used for the analyses.

## 3 RESULTS

The sample of 27 patients included 15 males (55.6%) and 12 females (44.4%) and comprised different stages of the disease. Mean age was 71.4±11 years, ranging from 43 to 88 years. Mean FVC was 2.23±0.74 L, ranging from 1.08 L to 3.63 L. Mean % of predicted FVC was 72.6±22%, from 45% to 93%.

The logistic regression models employed in this study were not capable of predicting PH, either likely or combined analysis of possible + likely PH. Table **1** shows the odds ratios (OR) for each predictor variable analyzed (T90, T88, PaO_2_, and DA-aO_2_).

Along the same lines, only a weak and non-significant correlation was observed for T90, T88, PaO_2_, and DA-aO_2_ with SPAP. SPAP could not be estimated in one patient in whom tricuspid regurgitation was absent, and thus 26 patients were included in this analysis. Pearson’s coefficients for the correlation between SPAP and hypoxia variables were as follows: -0.197 (*p* = 0.33) for PaO_2_; 0.099 (*p* = 0.62) for DA-aO_2_; 0.191 (*p* = 0.35) for T90; and 0.157 (*p* = 0.44) for T88.

Despite the inability of hypoxia data to predict PH and the lack of correlation with SPAP values, the results show that 24-h hypoxia levels were higher than expected considering resting PaO_2_ measurements. Table **2** shows T90 and T88 values according to hypoxia severity. Fig. (**1A** and **B**) emphasize these individual values in a scatterplot. It should be noted that even in patients with borderline normal PaO_2_, SpO_2_ below 90% was detected during a period corresponding to 17% of the day, that is, more than 4 h. This hypoxia duration was higher in groups with lower PaO_2_, with statistically significant differences. Indeed, resting PaO_2_ had a negative and moderate correlation with T90 (r = -0.398; *p* = 0.04), but not with T88 (r = -0.351; 0.07). None of the patients presented T90 or T88 of zero, although six patients of the 15 classified in group C (PaO_2_ = 70 mmHg) had T90 < 2%.

The analysis of sleep and wake periods revealed higher T90 and T88 during sleep, however without statistical significance. In six patients, we were unable to adequately discriminate between sleep and wake periods, and thus 21 patients were included in this analysis. Mean T90 was 30.7% and 37.1% during wake and sleep periods respectively (*p* = 0.08). Mean T88 was 18.2% and 22.6% during wake and sleep periods respectively (*p* = 0.18). Hemoglobin saturation declined at a slightly higher hourly rate during the wake period, 11.6 vs. 10.9 during sleep (*p* = 0.63).

## 4 DISCUSSION

The present results did not reveal a correlation between degree of hypoxia and PH. In fact, none of the hypoxia variables analyzed was capable of predicting PH, and there was no relevant correlation between hypoxia variables and SPAP. These data support the notion that other pathogenic mechanisms are probably involved the development of PH in IPF patients.

Hypoxia may induce PH through vasoconstriction or structural vascular changes, *i.e.* remodeling. Several alterations have been described in this situation, affecting arteries, arterioles and venules and destroying the capillary bed. Thickening of the adventitia has been described as a result of increased deposition of extracellular matrix proteins with accumulation of fibroblasts and myofibroblasts. Intima-media thickening may also be caused by smooth muscle hypertrophy and hyperplasia, in addition to collagen and elastin deposition. Muscularization of distal lung arterioles, a known feature of hypoxia-induced remodeling, has also been described. In addition, intimal hyperplasia has been described in IPF patients, together with fibrosis and duplication of the elastic lamina typical of small muscular pulmonary arteries [15].

Nevertheless, until the present moment, there is no significant evidence of morphological differences between the structural vascular changes affecting patients with PH induced by hypoxia or other reasons. Therefore it is not possible to affirm that other mechanisms besides hypoxia are implicated in PH in patients with IPF based solely on histopathological features.

Beyond these structural changes, a remarkable functional finding reported in the literature is the lack of correlation between lung volumes and PH [16]. One possible explanation for that would be the lack of a direct link between PH development and the extent of interstitial remodeling in IPF. Considering additional mechanisms of PH other than hypoxia, the dissociation between fibrosis and PH could be explained, at least in part, by endothelial dysfunction [16].

When looking at the biochemical changes related to hypoxia, it is possible to affirm that hypoxia and inflammation are inseparably intertwined [17]. If on the one hand hypoxia triggers the appearance of mediators that will ultimately lead to PH, on the other hand it is possible to speculate that such mediators constitute a physiological pathway that is shared with other triggering factors stemming from the inflammatory and/or fibrogenic process.

In this sense, several mediators have been implicated in both IPF and idiopathic pulmonary arterial hypertension [18]. For example, an increase in the production of profibrogenic leukotrienes has been reported in both conditions [19, 20]. This in turn causes an elevation in tumor necrosis factor alpha and in platelet- and fibroblast-derived growth factors, which are involved in vascular remodeling and lung fibrosis. A reduction in prostaglandin E2 levels has also been described, which may lead to further collagen deposition in the pulmonary interstitium and to further vascular remodeling [19].

Still regarding mediators, there is evidence of elevated serum levels of endothelin-1 in patients with IPF [21], as well as in patients with idiopathic pulmonary arterial hypertension [22]. Endothelin-1 is a potent vasoconstrictor that induces smooth muscle cell activity.

Nevertheless, the evidence regarding these shared mediators is not sufficient to produce a consistent explanatory model for the pathogenesis of PH. Also, it is not possible to assume that patients with IPF will inevitably develop PH. A large number of these patients do not have PH; and whereas treatment with bosentan, an endothelin-1 antagonist, was effective to treat idiopathic pulmonary arterial hypertension, it did not produce improvement in patients with IPF and PH [23].

In the present study, the cross-sectional design prevented us from analyzing the long-term impact of hypoxia duration in each patient. Nevertheless, our data show hypoxia occurring in connection with activities of daily living even in patients with resting PaO_2_ > 70 mmHg. This suggests the presence of hypoxia early on in IPF.

Based on our data, it is possible to speculate that the duration of disease in the presence of hypoxia may be more important than the intensity of hypoxia to predict PH. However, hypoxia is likely not the only mechanism triggering PH. There are no epidemiological data linking PH to slower development of IPF, a situation which would imply longer time of exposure to hypoxia.

Indirect evidence contrary to hypoxia as a pathogenic factor in PH and IPF have been obtained by Pouwels-Fry *et al.* [24], who observed that oxygen therapy does not interfere with the increase in SPAP during exercise in patients with IPF. In a different scenario, Blanco *et al.* [25] studied the effect of nitric oxide administration during rest and exercise in patients with IPF and observed a reduction in mean pulmonary artery pressure in both states, without significant changes in PaO_2_ or in ventilation/perfusion distributions. Differently from Pouwels-Fry *et al.*, these authors found a strong and significant correlation between mean pulmonary artery pressure and 6-keto-prostaglandin-F_1α_, an endothelium-derived molecule.

Regarding sleep and wake periods, hypoxia duration (T88 and T90) was slightly higher during sleep, even though the comparison was not statistically significant. This was probably due to the hypoventilation that occurs during sleep even in normal individuals [26]. No significant differences were observed between hourly hemoglobin desaturation index during sleep vs. wake period. Even though this finding is difficult to interpret (since during wake periods the effort associated with activities of daily living is a clear additional influence), it does not support the notion of severe respiratory sleep disturbances in this sample of patients.

## CONCLUSION

T90 and T88 measures in 24 hours reflect hypoxia in patients with borderline-normal PaO_2_. However, based on the present results, these variables are not useful to predict PH in IPF. The same was true for PaO_2_ and DA-aO_2_.

## Figures and Tables

**Fig. (1):**
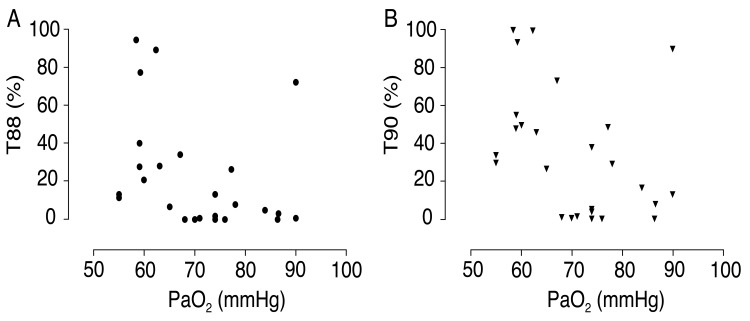
Percent time with SpO_2_ below 88% (T88) in 24 hours according to PaO_2_.

**Table 1:** Ability of hypoxia variables to predict pulmonary hypertension.

	**Pulmonary hypertension**			
	**Combined analysis of possible+ likely** **(*n* = 12)**		**Likely** **(*n* = 5)**	
	**OR**	***p***	**OR**	***p***
T90 (%)	1.005 (0.982 – 1.029)	0.66	1.014 (0.986 – 1.043)	0.31
T88 (%)	1.004 (0.978 – 1.031)	0.77	1.008 (0.97 – 1.040)	0.61
PaO_2_ (mmHg)	0.979 (0.910 – 1.054)	0.57	0.945 (0.853 – 1.048)	0.28
DA-aO_2_ (mmHg)	1.008 (0.931 – 1.091)	0.84	1.001 (0.905 – 1.107)	0.99

**Table 2:** Comparison of percent hypoxia time and systolic pulmonary artery pressure in patients with different degrees of resting PaO_2_.

	**PaO_2_**			
	**55-55.9 mmHg** **(*n* = 6)**	**60-69.9 mmHg** **(*n* = 6)**	**≥ 70 mmHg** **(*n* = 15)**	***p***
T90^a^	59.9±29	49.3±34	17.1±25	< 0.01
T88^b^	44.1±34	29.9±31	8.8±18	0.02
SPAP (mmHg)	47±31	36±20	35±10	0.38

^a^ % time with SpO_2_ < 90% in 24 h.

^b^ % time with SpO_2_ < 88% in 24 h.
